# Comparative study of reproductive and estrus characteristics of sexed and conventional semen in crossbred cows under field conditions

**DOI:** 10.14202/vetworld.2024.1119-1123

**Published:** 2024-05-17

**Authors:** Akshay Sharma, Pankaj Sood, Pravesh Kumar, Pururava Sharma, Ankit K. Ahuja, Vijender Negi, Harish Kumar, Amit K. Sharma

**Affiliations:** Department of Veterinary Gynecology and Obstetrics, Dr. GC Negi College of Veterinary and Animal Sciences, CSK Himachal Pradesh Agricultural University, Palampur, Himachal Pradesh, India

**Keywords:** conception rate, crossbred cows, estrus duration, pregnancy losses, sexed semen

## Abstract

**Background and Aim:**

Sexed semen (SS), a reproductive biotechnology tool, can alter the sex ratio of offspring in bovines. This study elucidates a comparative analysis of estrus-related parameters influencing conception rate and pregnancy losses under field conditions between conventional and SS.

**Materials and Methods:**

In the present study, artificial insemination with (SS; n = 143) and conventional semen (CS; n = 143) was performed at spontaneous estrus, i.e., 16–18 h after the onset of estrus signs, to analyze their comparative evaluation in terms of conception rates in crossbred cows under field conditions. Different parameters such as age, parity, body condition score (BCS), estrus duration, inter-estrus interval (IEI), diameter of pre-ovulatory follicle (DPOF) at estrus, and cervical mucus properties (pH and spinnbarkeit [SBK]) were recorded for each cow.

**Results:**

The first insemination conception rates for sexed and conventional semen were 55.24% and 63.63% whereas the overall conception rates were 49.14% and 57.37% on days 35 and 75 post-insemination, respectively, with no significant difference (p > 0.05). Conception rates between sexed and CS inseminations were statistically significant (p < 0.01), whereas factors such as age, parity, BCS, DPOF, IEI), and SBK value exhibited no substantial variance (p > 0.05) for both types of semen straw.

**Conclusion:**

SS straws yielded a conception rate equivalent to CS straws, with estrus duration being the key factor affecting conception under field conditions.

## Introduction

Sperm sexing is an essential reproductive technology that governs the sex ratio of offspring in the dairy sector [1]. In India, sexed semen (SS) use has become quite essential under field conditions due to the limited expansion of dairy herds and the percentage of progressive farmers [2]. One of the main benefits of using SS is an increase in birth rate of female calves (approximately 70-80%) and occasionally up to 90% [3], which significantly affects the profitability of the dairy enterprise and ensures adequate heifers and pregnancies for lactation [4].

SS has less efficacy due to a lower concentration of spermatozoa compared to conventional semen (CS) straws [4]. As a result, the likelihood of conception in heifers [5] and cows [6] through artificial insemination (AI) with SS is approximately 70%–80% to that of CS, which is notably higher. Various physiological factors that could affect the conception rate following AI with conventional and SS have been studied [1, 7]; however, information on each factor is limited. Pregnancy losses with low fertility following insemination with SS are mainly attributed to impaired sperm function caused by fluorochrome and exposure to ultraviolet laser beam, which alters DNA integrity [5]. On the other hand, pre-capacitated spermatozoa often plummet conception and may lead to poor embryo quality [8]. To overcome these negative effects, several techniques, such as altering the sperm concentration in straw [7], fixed-time AI [9], AI at spontaneous estrus [10], and intracorneal semen deposition [11], have been employed. To effectively use SS, the selection of cows is a critical factor.

Thus, this study aimed to compare the analysis of estrus-related parameters influencing the conception rate and pregnancy losses between conventional and SS.

## Materials and Methods

### Ethical approval

The absence of invasive procedures and the focus on routine diagnostics tended to no requirement of ethical committee approval.

### Study period and location

The study was conducted between June 2021 to January 2023 in Palampur, Himachal Pradesh, India.

### Experimental animals and design

The study population consisted of healthy crossbred cows (75% Jersey blood and 25% red Sindhi blood) of the North-West Himalayan region (32.1109°N and 76.5363°E, respectively) reared in a loose housing system and fed 3 times a day as recommended by the National Research Council (2001). Recording of various parameters of cows such as parity (<4), age (2.0–6.5 years), body condition score (BCS) (2.5–3.25), estrus duration, and inter-estrus interval (IEI) (18–24 days) was performed. The experiment was designed to determine the efficacy of sexed and CS straws at spontaneous estrus in crossbred cows randomly allocated to (SS, n = 143) and (CS, n = 143) groups under field conditions.

### Detection of estrus and insemination

Visual signs of estrus were detected twice a day for 30 min (before milking around 05:00 h and at the end of the afternoon 17:00 h), and signs such as mucous vulvar discharge, cajoling (Flehman’s reaction), restlessness, vulvar sniffing of another cow, chin resting, attempt to mount, mounting head side, and stand to be mounted were observed. The cows in estrus were appropriately secured in a crush before transrectal examination and sonography (TRUS; Mindray Z5 VET; Model 2017; Mindray, Shenzen, China). Both the transducer and the sleeved arm were thoroughly lubricated with an obstetric lubricant to enable painless and easy insertion through the anal sphincter. The transducer was placed proximal to the genital tract and moved slowly toward the cranial side to visualize the ovarian structures, i.e., the diameter of the preovulatory follicle.

We cleaned the vulvar region of cows with a highly tonic-contractile uterus and aspirated the genital discharge of the cow using the AI sheath. The pH of the genital discharge was immediately evaluated using pH paper (range, 6.0–8.0), and the spinnbarkeit (SBK) value was determined using the collected cervical mucus. Two to three drops of the collected mucus sample were placed on a grease-free slide, and another slide was placed over it. The mucus was stretched between the two slides by moving the second slide away from the first slide until it broke. A plastic geometrical scale (cm) was used to measure the distance between the two slides immediately before the breakage of the mucus string.

AI with SS and CS was performed in cows with clear genital discharge. Both groups were inseminated after 16–18 h (mid-estrus) of commencement of visual signs of estrus with placement of sperm suspension in the uterine body. We randomly allocated cows for AI with French mini straw containing SS (IntelliGen technology Sexcel semen straw; GLBX SEXED ZIG JY PAT 29JE4235 (1) 9761 containing 2 million spermatozoa) and CS (Himachal Pradesh Livestock Development Board; PLM-JYCB-117 JY X SW SS-Palampur 070522 (1) 4116 containing 20 million spermatozoa) for AI. Live spermatozoa and progressive motility were 58%, 55%, and 82%, 78%, respectively. The standard thawing procedure recommended by the Centre for Agriculture and Bioscience International was adopted. Straws were thawed in a Cito-thaw water bath at 37°C for 30 s.

### Pregnancy outcome

Gynecological examination for pregnancy outcome was performed using transrectal sonography. For assessment of late embryonic mortality, the examination was carried out scrupulously every 72 h from non-return to estrus until the onset of period of the fetus. Thereafter, the cows were assessed by rectal palpation on 75-day post-insemination for evidence of pregnancy. The sex of the newborn calf was recorded at calving.

### Statistical analysis

Numeric data for all parameters are expressed as mean ± standard error of the mean, and statistical analyses were performed using Welch’s t-test and Pearson’s Chi-square test (for categorical data). Pearson’s correlation coefficient was calculated to determine the correlation between different animal and estrus-related parameters recorded in the present study. Statistical analysis was performed using Statistical Package for the Social Sciences version 21.0 (IBM Corp., NY, USA).

## Results

No significant difference (p < 0.05) was observed in the conception rate, overall conception rate, and overall pregnancy rate after insemination with sexed and CS on day 35 (Table-1). The impact of various animal and estrus-related parameters is summarized in Table-2. Milk yield, estrus duration, and diameter of pre-ovulatory follicle in cows significantly influenced conception following insemination with sexed and CS (p < 0.01). Interestingly, an increase in milk yield per day and estrus duration negatively affected conception rate whereas DPOF (mm) negatively affected conception in cows inseminated with SS compared to CS.

**Table 1:** Comparison of reproductive indices in sexed and conventional semen.

Parameters	Sexed semen	Conventional semen	p-value
35-day first insemination conception rate (%)	55.24 (79/143)	63.63 (91/143)	0.09
Overall conception rate (%)	49.14 (143/291)	57.37 (140/244)	0.08
Pregnancy losses (%)	6.99 (10/143)	4.20 (6/143)	0.14
Overall pregnancy rate (%)	46.83 (133/284)	56.77 (134/236)	0.06
Female calves born (%)	90.90 (120/132)	47.76 (64/134)	0.00

**Table 2:** Comparison of animal and estrus-related variables affecting conception in sexed and conventional semen.

Parameters	Sexed semen	conventional semen	p-value
Animal-related parameters			
Age (years)			
m ± SEM	4.9 ± 0.23	5.8 ± 0.19	0.003
Median	4.8	5.8	
CI	4.4–5.3	5.43–6.17	
Parity (nos.)			
m ± SEM	1.46 ± 0.10	1.66 ± 0.09	0.153
Median	1	2	
CI	1.26–1.65	1.48–1.84	
Body condition score (1–5)			
m ± SEM	2.60 ± 0.01	2.59 ± 0.01	0.163
Median	2.6	2.6	
CI	2.58–2.62	2.57–2.60	
Milk Yield (liters)			
m ± SEM	7.89 ± 0.57	5.82 ± 0.37	0.002
Median	7.0	6.0	
CI	6.77–9.00	5.09–6.55	
Estrus-related parameters			
Estrus duration (h)			
m ± SEM	28.71 ± 1.11	27.77 ± 0.65	0.002
Median	27.0	26.0	
CI	26.53–30.89	26.50–29.04	
Inter-estrus interval (days)			
m ± SEM	20.35 ± 0.13	20.16 ± 0.12	0.283
Median	21.0	20.0	
CI	20.10–20.61	19.93–20.40	
Diameter of pre-ovulatory follicle (mm)			
m ± SEM	13.71 ± 0.13	13.83 ± 0.08	0.007
Median	13.6	14.1	
CI	13.46–13.97	13.67–13.99	
pH			
m ± SEM	7.61 ± 0.02	7.61 ± 0.02	0.684
Median	7.6	7.6	
CI	7.57–7.65	7.57–7.65	
Services per conception (nos.)			
m ± SEM	1.64 ± 0.08	1.50 ± 0.62	0.150
Median	1	1	
CI	1.48–1.80	0.29–2.71	

SEM=Standard error of the mean, CI=Confidence interval

Furthermore, Pearson’s Chi-square analysis corroborated that only estrus duration had a significant role in successful conception (SS; χ^2^ = 33.349 vs. CS; χ^2^ = 15.220; p < 0.0001), whereas parity, BCS, IEI, SBK, and pH of genital discharge did not affect the eventual fate (Table-3).

**Table 3:** Effect of semen type, age, parity, BCS, estrus duration, inter-estrus interval, diameter of pre-ovulatory follicle, and spinnbarkeit values on conception using sexed and conventional semen.

Factor	Df	Chi-square (p-value)		

		Cumulative	Sexed semen	Conventional semen
Semen type	1	3.172 (0.075)		
Age	2	2.790 (0.248)	0.996 (0.608)	2.622 (0.270)
Parity	3	3.698 (0.296)	6.230 (0.101)	0.748 (0.862)
BCS	2	3.659 (0.161)	3.749 (0.153)	2.510 (0.285)
Estrus duration	2	44.849 (0.000)	33.349 (0.000)	15.220 (0.000)
Inter-estrus interval	2	0.178 (0.915)	2.211 (0.331)	0.760 (0.684)
DPOF	2	1.794 (0.408)	1.451 (0.484)	1.263 (0.532)
Spinnbarkeit	2	2.320 (0.313)	0.417 (0.812)	5.697 (0.058)

BCS=Body condition score, DPOF=Diameter of pre-ovulatory follicle

Pearson correlation analysis confirmed a strong association between different variables affecting SS and CS conception. A significant positive correlation was observed between parity, BCS, and IEI for sexes and CS (Table-4). However, the present study corroborated the relationship between estrus duration and conception with SS as a significant negative correlation (r = 0.54; p < 0.01) compared with that to CS (r = 0.26; p < 0.01) and eventually summarized the myth associated with low conception rate with SS under field conditions. Furthermore, the percentage of female calves born was significantly higher (91.09 vs. 47.76; p < 0.01) with sexed than with CS.

**Table 4:** Correlation matrix depicting relationship between different variables in sexed and conventional semen-inseminated cows.

Semen	Parameters	Age	Parity	BCS	Estrus duration	Inter-estrus interval	Pre-ovulatory follicle size	Conception
SS	Age	1	0.839**	0.235*	0.200*	0.295**	0.139	−0.102
CS			0.875**	−0.233**	0.230**	0.243**	-0.048	0.058
SS	Parity		1	0.306**	0.331**	0.279**	0.165	−0.197*
CS				−0.223**	0.317**	0.241**	−0.106	−0.011
SS	BCS			1	0.199*	0.058	0.105	−0.165
CS					−0.023	−0.014	0.054	0.004
SS	Estrus duration				1	0.116	0.007	−0.540**
CS						0.036	−0.184*	−0.260**
SS	Inter-estrus interval					1	0.031	−0.082
CS							0.043	0.03
SS	DPOF						1	0.009
CS								0.089
SS	Conception							1
CS								

BCS=Body condition score, DPOF=Diameter of pre-ovulatory follice, SS=Sexed semen, CS=Conventional semen

## Discussion

To effectively manage the sex ratio of offspring and allow genetic improvement of existing cattle stocks at field level, AI of SS must be performed. However, the main concern associated with the limited use of SS is the average conception rate compared to that of CS [12]. Comprehending the difference between sexed and CS can help attain steady conception rates after AI using SS. Therefore, the aim of the current study was to determine the conception outcome with sexed and CS during spontaneous estrus in relation to different animal characteristics and estrus parameters.

Many studies have cited [7, 10–12] varying conception rates with SS, highlighting the importance of farm-related factors; however, there is sparse literature regarding field conditions. Similarly, in the present study, several factors impact conception rate including age, body condition, parity, and precision of estrus detection [13]. In general, the difference in conception rates between sexed and CS ranges from 10% to 30% [14, 15], which is primarily a function of low sperm dosage [10, 16]. A connection has been proposed between sex-sorted sperm and a higher incidence of embryonic death that could decrease the conception rate [13]. Nonetheless, some field studies have documented the first insemination conception rate with SS to be above 50% [15, 17]. However, it was still lower than the CS, probably due to embryonic mortality [13]; however, this was not the case in the current study, as there were no significant differences in pregnancy losses between the two types of semen.

Although the conception rate for sexed and CS has been fairly superior for heifers, they are naturally more fertile than lactating cows [17], as documented in the current study. Reduced pregnancy per AI in multiparous cows is mainly attributed to differences in milk production, energy balance, postparturient diseases, mastitis, and lameness [18].

Significant variations in the duration of estrus and the time interval between its onset, end, and the time of ovulation have been associated with the conception rate with sexed and CS in dairy cows [19]. A thorough exploration of the literature showed that the duration of estrus ranged from 7 to 16 h, and the onset and end of estrus to ovulation time ranged from 27 to 30 h and 12 to 19 h, respectively [20]. Therefore, determining the optimal AI timing with conventional and SS does not hold proximal similarity; thus, SS-based AI programs remain a major challenge [21].

Extending the use of SS for improvement in milk production, various authors have devised strategies to maximize the number of female calves in large-scale dairy farms, which have been documented in the literature. The birth rate of female calves with SS varies between 81% and 92% [13], which is similar to the present study. However, some studies have reported similar [22] and contrasting outcomes [23, 24] with the introduction of SS in dairy cows. However, the use of SS under field conditions has a number of shortcomings, such as different factors affecting the rate of conception, the return of female calves, and the suitability for different breeds of cattle.

## Conclusion

SS increases the number of replacement heifers accessible to dairy farmers by enhancing their herd reproductive efficiency, facilitating gender-based herd management in crossbred cows; nevertheless, it is ideal to utilize SS in younger heifers or in cows with low parity with optimal BCS to maximize conception rates and return on investment. In peroration, SS yielded a conception rate equivalent to that of CS, with estrus duration emerging as a critical factor influencing conception success. The practical application of SS requires careful consideration of a wide range of estrus and animal-related parameters to determine the optimal fertility rate.

## Authors’ Contribution

AS and PS: Conceptualization. PK: Methodology. AKS: Validation. AKA: Formal analysis. PuS, VN, and HK: Investigation. AS, PuS, and PK: Data curation, AS, PuS, and AKA: drafted and revised the manuscript, All authors have read, reviewed, and approved the final manuscript.

## References

[1] Oikawa K, Yamazaki T, Yamaguchi S, Abe H, Bai H, Takahashi M, Kawahara M (2019). Effects of use of conventional and sexed semen on the conception rate in heifers:A comparison study. Theriogenology.

[2] Boneya G (2021). Sexed semen and major factors affecting its conception rate in dairy cattle. Int. J. Adv. Res. Biol. Sci.

[3] De Vries A, Overton M, Fetrow J, Leslie K, Eicker S, Rogers G (2008). Exploring the impact of sexed semen on the structure of the dairy industry. J. Dairy Sci.

[4] Stevenson J.S, Ahmadzadeh A (2011). Replacement management in cattle breeding standards and pregnancy management. Encyclopedia of Dairy Sciences.

[5] Healy A.A, House J.K, Thomson P.C (2013). Artificial insemination field data on the use of sexed and conventional semen in nulliparous Holstein heifers. J. Dairy Sci.

[6] Norman H.D, Hutchison J.L, Miller R.H (2010). Use of sexed semen and its effect on conception rate, calf sex, dystocia, and stillbirth of Holsteins in the United States. J. Dairy Sci.

[7] DeJarnette J.M, Nebel R.L, Marshall C.E (2009). Evaluating the success of sex-sorted semen in US dairy herds from on-farm records. Theriogenology.

[8] Pirez M.C, Steele H, Reese S, Kolle S (2020). Bovine sperm-oviduct interactions are characterized by specific sperm behavior, ultrastructure, and tubal reactions, which are impacted by sex sorting. Sci. Rep.

[9] Chebel R.C, Cunha T (2020). Optimization of timing of insemination of dairy heifers inseminated with sex-sorted semen. J. Dairy Sci.

[10] Reese S, Pirez C.M, Steele H, Kolle S (2021). The reproductive success of bovine sperm after sex-sorting:A meta-analysis. Sci. Rep.

[11] Andersson M, Taponen J, Kommeri M, Dahlbom M (2006). Pregnancy rates in lactating Holstein-Friesian cows after artificial insemination with sexed sperm. Reprod. Domest. Anim.

[12] Maicas C, Hutchinson I.A, Kenneally J, Grant J, Cromie A.R, Lonergan P, Butler S.T (2019). Fertility of fresh and frozen sex-sorted semen in dairy cows and heifers in seasonal calving pasture-based herds. J. Dairy Sci.

[13] Seidel G.E, Schenk J.L (2008). Pregnancy rates in cattle with cryopreserved sexed sperm:Effects of sperm numbers per inseminate and site of sperm deposition. Anim. Reprod. Sci.

[14] Seidel G.E, Schenk J.L, Herickhoff L.A, Doyle S.P, Brink Z, Green R.D, Cran D.G (1999). Insemination of heifers with sexed sperm. Theriogenology.

[15] Borchersen G, Peacock M (2009). Danish A.I. field data with sexed semen. Theriogenology.

[16] Frijters A.C.J, Mullaart E, Roelofs R.M.G, Van Hoorne R.P, Moreno J.F, Moreno O, Merton J.S (2009). What affects fertility of sexed bull semen more, low sperm dosage or the sorting process?. Theriogenology.

[17] Garner D.L, Seidel G.E (2008). History of commercializing sexed semen for cattle. Theriogenology.

[18] Santos J.E.P, Cerri R.L.A, Ballou M.A, Higginbotham G.E, Kirk J.H (2004). Effect of timing of first clinical mastitis occurrence on lactational and reproductive performance of Holstein dairy cows. Anim. Reprod. Sci.

[19] Lopez-Gatius F (2022). Revisiting the timing of insemination at spontaneous Estrus in dairy cattle. Animals.

[20] Dransfield M.B.G, Nebel R.L, Pearson R.E, Warnick L.D (1998). Timing of insemination for dairy cows identified in estrus by a radiotelemetric Estrus detection system. J. Dairy Sci.

[21] Sales J.N.S, Neves K.A.L, Souza A.H, Crepaldi G.A, Sala R.V, Fosado M, Campos Filho E.P, De Faria M, Sa Filho M.F, Baruselli P.S (2011). Timing of insemination and fertility in dairy and beef cattle receiving timed artificial insemination using sex-sorted sperm. Theriogenology.

[22] Patra M.K, Sasidharan J.K, Rajput A.S, Sharma R, Reza M.R.A, Das G.K, Tomar A.K.S, Ghosh S.K, Gaur G.K (2023). Evaluation of sexed semen-based artificial insemination in Tharparkar cattle under organized farm condition. Reprod. Domest. Anim.

[23] Patel S.B, Jethva P.C (2019). Use of sexed semen in Indian dairy cattle:A case study. Indian J. Vet. Sci. Biotech.

[24] Joshi S, Bhave K, Potdar V, Gaundare Y, Punde N, Shirsath T, Swaminathan M (2021). Performance of sex-sorted semen under Indian smallholder dairy farming systems. Int. J. Curr. Microbiol. Appl. Sci.

